# Supplementary material for: Transcriptional Landscape of 3D vs. 2D Ovarian Cancer Cell Models
Source: Cancers (Basel). 2023 Jun 26;15(13):3350. doi: 10.3390/cancers15133350 (PMC10340606; doi:10.3390/cancers15133350)
Supplement: Supplementary file 1 [file cancers-15-03350-s001.zip › cancers-2125248-supplementary.pdf]

Transcriptional landscape of 3D vs 2D ovarian cancer cell models

Rachel Kerslake[1], Birhanu Belay[2], Suzana Panfilov[1], Marcia Hall[1,3], Ioannis Kyrou[4,5,6,7,8], Harpal S. Randeva[4,5], Jari Hyttinen[2], Emmanouil Karteris[1*†] and Cristina Sisu[1*†]

Supplementary Materials

Table S1. Cell line information and associated accession codes.

Accession Number	SRA code	Cell line	Subtype	Condition	Scaffold
PRJNA472611	SRR7204219	A1847	Carcinoma	3D	Agarose
	SRR7204220	A1847	Carcinoma	2D	/
	SRR7204221	A2780	HGSOC	3D	Agarose
	SRR7204222	A2780	HGSOC	2D	/
	SRR7204223	OVCAR3	HGSOC	3D	Agarose
	SRR7204224	OVCAR3	HGSOC	2D	/
	SRR7204225	OVCAR4	HGSOC	3D	Agarose
	SRR7204226	OVCAR4	HGSOC	2D	/
	SRR7204227	OVCAR5	HGSOC	3D	Agarose
	SRR7204228	OVCAR5	HGSOC	2D	/
	SRR7204231	OVCAR10	HGSOC	3D	Agarose
	SRR7204232	OVCAR10	HGSOC	2D	/
	SRR7204233	OVCAR8	HGSOC	3D	Agarose
	SRR7204234	OVCAR8	HGSOC	2D	/
	SRR7204235	SKOV-3	HGSOC	3D	Agarose
	SRR7204236	SKOV-3	HGSOC	2D	/
	SRR7204237	PEO1	HGSOC	3D	Agarose
	SRR7204238	PEO1	HGSOC	2D	/
	SRR7204229	C30	Carcinoma	3D	Agarose
	SRR7204230	C30	Carcinoma	2D	/
	SRR7204242	C70	Carcinoma	3D	Agarose
	SRR7204241	C70	Carcinoma	2D	/
	SRR7204240	UPN275	MAC	3D	Agarose
	SRR7204239	UPN275	MAC	2D	/
PRJNA530150	SRR8823257	OVCAR8	HGSOC	2D	/
	SRR8823258	OVCAR8	HGSOC	2D	/
	SRR8823259	OVCAR8	HGSOC	2D	/
	SRR8823260	OVCAR8	HGSOC	2D	/
	SRR8823265	OVCAR8	HGSOC	3D	Matrigel

	SRR8823266	OVCAR8	HGSOC	3D	Matrigel
	SRR8823267	OVCAR8	HGSOC	3D	Matrigel
	SRR8823268	OVCAR8	HGSOC	3D	Matrigel
	SRR8823273	OVCAR8	HGSOC	2D	/
	SRR8823273	OVCAR8	HGSOC	2D	/
	SRR8823273	OVCAR8	HGSOC	2D	/
	SRR8823273	OVCAR8	HGSOC	2D	/
	SRR8823280	OVCAR8	HGSOC	3D	Matrigel
	SRR8823281	OVCAR8	HGSOC	3D	Matrigel
	SRR8823282	OVCAR8	HGSOC	3D	Matrigel
	SRR8823283	OVCAR8	HGSOC	3D	Matrigel
PRJNA564843	SRR10096845	OVCAR8	HGSOC	2D	/
	SRR10096844	OVCAR8	HGSOC	2D	/
	SRR10096843	OVCAR8	HGSOC	2D	/
	SRR10096841	OVCAR8	HGSOC	3D	Collagen
	SRR10096842	OVCAR8	HGSOC	3D	Collagen
	SRR10096840	OVCAR8	HGSOC	3D	Collagen
	SRR10096839	OVCAR4	HGSOC	2D	/
	SRR10096838	OVCAR4	HGSOC	2D	/
	SRR10096837	OVCAR4	HGSOC	2D	/
	SRR10096836	OVCAR4	HGSOC	3D	Collagen
	SRR10096835	OVCAR4	HGSOC	3D	Collagen
	SRR10096834	OVCAR4	HGSOC	3D	Collagen
	SRR10096828	Kuramochi	HGSOC	3D	Collagen
	SRR10096829	Kuramochi	HGSOC	3D	Collagen
	SRR10096830	Kuramochi	HGSOC	3D	Collagen
	SRR10096831	Kuramochi	HGSOC	2D	/
	SRR10096832	Kuramochi	HGSOC	2D	/
	SRR10096833	Kuramochi	HGSOC	2D	/
PRJNA232817	GSM1300206	IGROV-1	EAC	2D	/
	GSM1300207	IGROV-1	EAC	2D	/
	GSM1300208	IGROV-1	EAC	2D	/
	GSM1300209	IGROV-1	EAC	3D	Low Attachment
	GSM1300210	IGROV-1	EAC	3D	Low Attachment
	GSM1300211	IGROV-1	EAC	3D	Low Attachment
PRJNA318768	GSM2125384	HEY	HGSOC	2D	/
	GSM2125385	HEY	HGSOC	2D	/
	GSM2125386	HEY	HGSOC	2D	/
	GSM2125387	HEY	HGSOC	2D	/

	GSM2125388	HEY	HGSOC	3D	Hanging drop
	GSM2125389	HEY	HGSOC	3D	Hanging drop
	GSM2125390	HEY	HGSOC	3D	Hanging drop
	GSM2125391	HEY	HGSOC	3D	Hanging drop

Table S2. A) Top 150 differentially expressed genes in OVCAR8 in all three grown media: grown on agarose, collagen, Matrigel. The differential expression in each of the growth media is with respect to 2D controls of the OVCAR8;

**OVCAR8 v 2D controls**					
ACTB	CD70	EVL	LAPTM5	PAX8	SLPI
ADAMTS1	CDH13	FAM83A	LAYN	PDCD10	SNCA
ADAMTS6	CDH2	FBN2	LDHB	PDE1C	SNRPN
AEBP1	CDKN2A	FGFBP1	MAP1B	PDPN	SPAG4
AJAP1	CFB	FN1	MFSD2B	PHYHD1	SPANXB1
ALDOC	CLGN	GFRA1	MGMT	PI3	SPINT2
ANGPTL4	CLMP	GGT1	MMP1	PLIN2	SRGN
ANPEP	COL3A1	GREM1	MSLN	PLTP	ST20-MTHFS
ANXA8L1	COL7A1	GSTM3	MT1M	PPP1R14A	STMN3
ARMCX2	CRIP1	HBQ1	MTAP	PRAME	SUN3
B4GALNT4	CTCFL	HCLS1	MX1	PTGDS	TENM2
BCAT1	CTSF	HLA-DRB1	MYEF2	PTK7	TFPI2
BCL2A1	CXCL1	HSPB2	NACA2	PTPRS	TGFBI
BEX1	CXCL2	IFI27	NDN	RFTN1	THBS1
BEX4	CXCL5	IFI44	NDRG1	RPL7	TRIM58
BGN	CXCL8	IFI44L	NEFH	RSPO4	TSPAN1
BHLHE40	DDIT4	IFI6	NETO2	SAA1	TSPYL5
BNIP3	DKK 1.00	IFITM1	NEURL1	SCOC	TUSC3
BST2	DUSP23	IGFBP2	NMRAL1	SDC2	TYMP
C3	DZIP1	IGFBP3	NNMT	SEMA3C	UBB
C3orf14	EDARADD	IGFN1	NPPB	SERPINB2	UCHL1
CALB1	EFEMP2	IL1A	NPW	SLC38A5	UCP2
CALB2	EGR1	IL1B	NRG1	SLC39A4	ZNF699
CCL2	ELOVL2	IL6	OAS3	SLC6A15	ZNF83
CCL20	ESM1	LAMC2	P3H2	SLFN11	ZSCAN18

Table S2. B) Top 150 differentially expressed genes across the cell lines A1847, A2780, C30, C70, OVCAR3, OVCAR4, OVCAR5, OVCAR8, OVCAR10, PEO1, SKOV-3, UPN275 grown on agarose vs. 2D controls

**Differentially expressed genes in Agarose vs 2D controls**					
ADAMTS1	COL26A1	FOXD1	KLK8	NUPR1	SPANXB1
ADAMTS6	CP	GALNT3	KRT19	PAX8	SPARC
ADGRG6	CRB3	GBP1	KRT23	PDCD10	SPINT2
AKT3	CXCL1	GBP2	KRTCAP3	PLAT	SPON1
ANPEP	CXCL2	GDF15	LAD1	PLIN2	SPP1
ATP2B2	CXCL8	GJB2	LAMA3	PLTP	ST14
AXL	CYGB	GLDC	LAMC2	PPP1R14A	ST6GALNAC1
BEX1	CYP1A1	GLUL	LCN2	PRAME	TACSTD2
BMP7	DKK 1.00	GPNMB	LCP1	PROM1	TAGLN
BST2	ECM1	GSTM3	LDHB	PRSS22	TFPI2
C3	EDN2	HGD	LGALS13	PTGS1	TGFBI
CALB2	EHD2	IDO1	LGR5	RAB25	TMC4
CBLC	ELF3	IGFBP2	LY6K	S100A14	TMPRSS4
CCL2	EMX2	IGFBP3	MACROD2	S100A9	TNFRSF6B
CCL20	EPS8L1	IGFBP5	MAL2	SCGB2A1	TRIML2
CD70	ERP27	IGFBP7	MECOM	SCOC	TSPAN1
CD74	ESRP1	IL1B	MMP1	SDC2	TSTD1
CDH1	EYA2	IL1R1	MMP7	SGIP1	TUSC3
CDH6	FABP6	IL1R2	MPZL2	SLC17A9	UBB
CDKN2A	FAM83A	IL6	MUC16	SLC34A2	UCHL1
CHI3L1	FAT2	IL7R	NCAM1	SLC38A5	UQCRH
CLDN11	FKBP10	ITGB6	NETO2	SLC39A4	UQCRHL
CLDN4	FLNC	KCNC3	NPPB	SLPI	VTCN1
COL18A1	FN1	KISS1	NPTX2	SNRPN	WFDC2
COL23A1	FOSL1	KLK10	NRCAM	SOX17	WNT7A

Table S2. C) Top 150 differentially expressed genes in the cell lines Kuramochi, OVCAR4, and OVCAR8, grown on collagen vs. 2D controls or the respective cell lines.

**Top 150 differentially expressed genes in Collagen vs 2D controls**					
A2M	COL3A1	GNGT2	LAMA3	OXTR	SOX17
ABI3	CP	GOLIM4	LCN2	PDCD10	SPARC
ADGRG1	CRB3	ID4	LEMD1	PKP3	SPON1
AFAP1L2	CTCFL	IFI27	LGALS13	PLPP2	SPP1
AKT3	CYGB	IFI44	LGR5	PRSS22	ST14
APOE	DAPL1	IFI44L	LGR6	PTGS1	ST6GALNAC1
ARID4A	DCDC2	IFI6	LIPG	RAB25	STRA6
BAALC	DKK 1.00	IFITM1	LRRN2	RAPGEF3	SULF1
BHLHE41	DPEP3	IGF2	LY6K	REC8	SYNE4
BMP7	EDN2	IGFBP2	LYPD1	RHOD	TACSTD2
BOC	ELF3	IGFBP5	MAL2	RNF212	TFPI2
C3	EMX2	IGFBP7	MECOM	S100A1	TGFBI
CBLC	EPB41L3	IGFL1	MLPH	S100A14	THY1
CCDC146	EPCAM	IL18	MMP7	S100A4	TMC4
CCL2	EPS8L1	IL1R2	MPZL2	S100A9	TNC
CDA	ESRP1	IL7R	MUC16	SC5D	TNFRSF6B
CDH1	FABP3	ITGB3	MX1	SCGB2A1	TRIM58
CDH6	FBXO2	ITGB6	MX2	SELENBP1	TSPYL5
CFI	FKBP10	KCNC3	MYH7B	SFN	TSTD1
CLDN16	FLNC	KISS1	NDN	SFTA2	TUSC3
CLDN4	FN1	KLK8	NEFH	SLC17A9	UBB
CLDN7	FOXD1	KRT19	NKAIN4	SLC34A2	UQCRH
CNTN1	FXYD6	KRT7	NPTX2	SLC38A5	UQCRHL
COL1A2	GDF15	KRTCAP3	NRCAM	SLPI	WFDC2
COL26A1	GLUL	LAD1	NXPH2	SMIM22	WNT7A

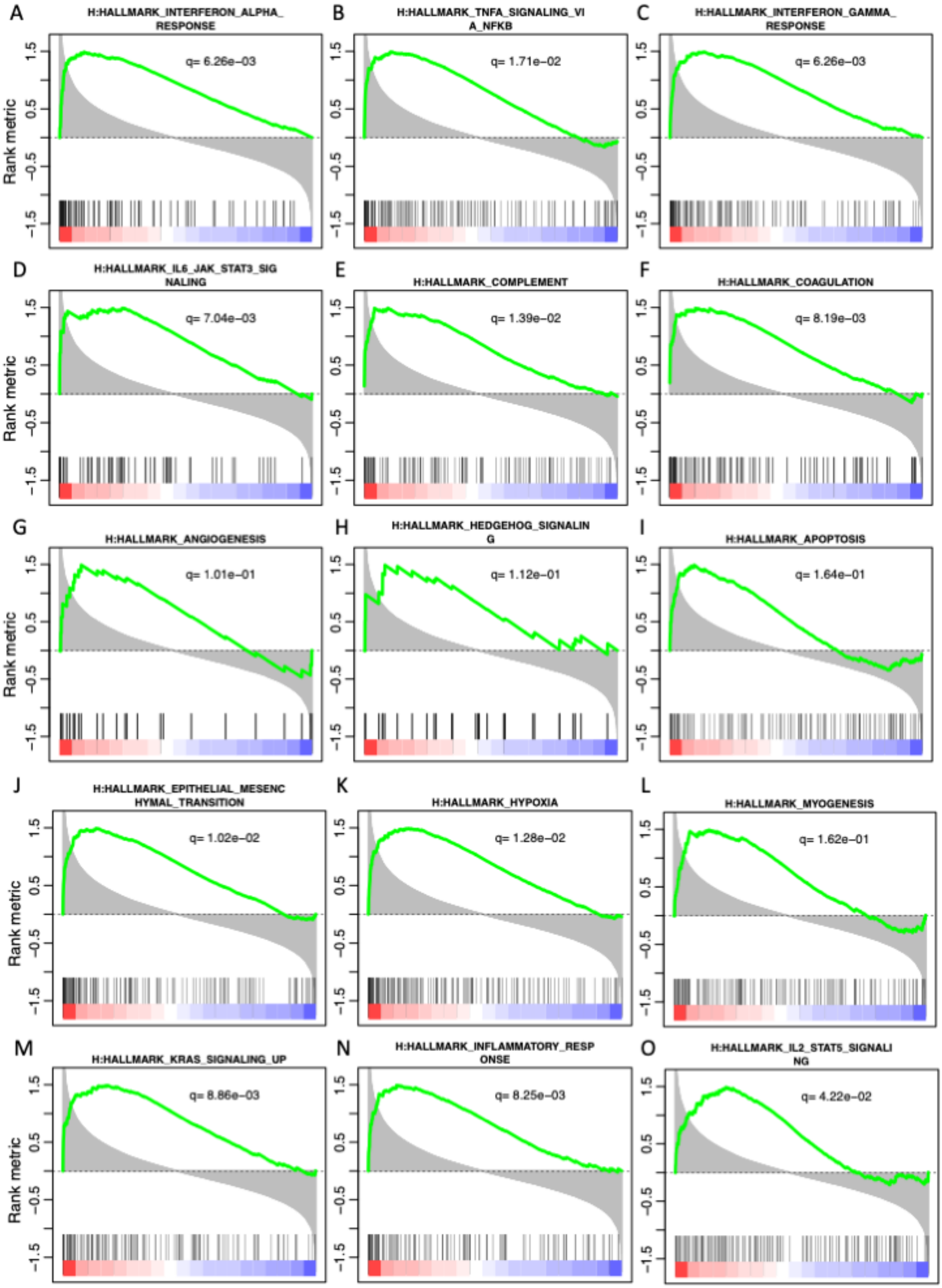
A
H:HALLMARK_INTERFERON_ALPHA_RESPONSE
q= 6.26e−03
B
H:HALLMARK_TNFA_SIGNALING_VIA_NFKB
q= 1.71e−02
C
H:HALLMARK_INTERFERON_GAMMA_RESPONSE
q= 6.26e−03
D
H:HALLMARK_IL6_JAK_STAT3_SIGNALING
q= 7.04e−03
E
H:HALLMARK_COMPLEMENT
q= 1.39e−02
F
H:HALLMARK_COAGULATION
q= 8.19e−03
G
H:HALLMARK_ANGIOGENESIS
q= 1.01e−01
H
H:HALLMARK_HEDGEHOG_SIGNALING
q= 1.12e−01
I
H:HALLMARK_APOPTOSIS
q= 1.64e−01
J
H:HALLMARK_EPITHELIAL_MESENCHYMAL_TRANSITION
q= 1.02e−02
K
H:HALLMARK_HYPOXIA
q= 1.28e−02
L
H:HALLMARK_MYOGENESIS
q= 1.62e−01
M
H:HALLMARK_KRAS_SIGNALING_UP
q= 8.86e−03
N
H:HALLMARK_INFLAMMATORY_RESPONSE
q= 8.25e−03
O
H:HALLMARK_IL2_STAT5_SIGNALING
q= 4.22e−02
Rank metric
1.5
0.5
−0.5
−1.5

Figure S1. Top Enriched Gene sets for 2D vs. 3D OVCAR8. Combined panel of enrichment curves showing processes associated with cancer hallmarks. The GSEA was performed by comparing the expression profiles in the 2D and 3D cultures and highlights differential expression patters of the genes associated with various cancer hallmarks. **(A)**, Interferon Alpha Response; **(B)**, TNF-a signalling; **(C)**, Interferon Gamma Response; **(D),** IL-6-JAK-STAT3 signalling; **(E)**, Complement; **(F)**, Coagulation; **(G)**, Angiogenesis; **(H)**, Hedgehog signalling; **(I)**, Apoptosis; **(J),** Epithelial Mesenchymal Transition; **(K)**, Hypoxia; **(L)**, Myogenesis; **(M)**, KRAS signalling; **(N)**, Inflammatory Response; **(O)**, IL-2 STAT3 signalling and. Black vertical bars represent gene rank using shorted list metric. Green curve corresponds to "running statistics" of the enrichment score (ES).

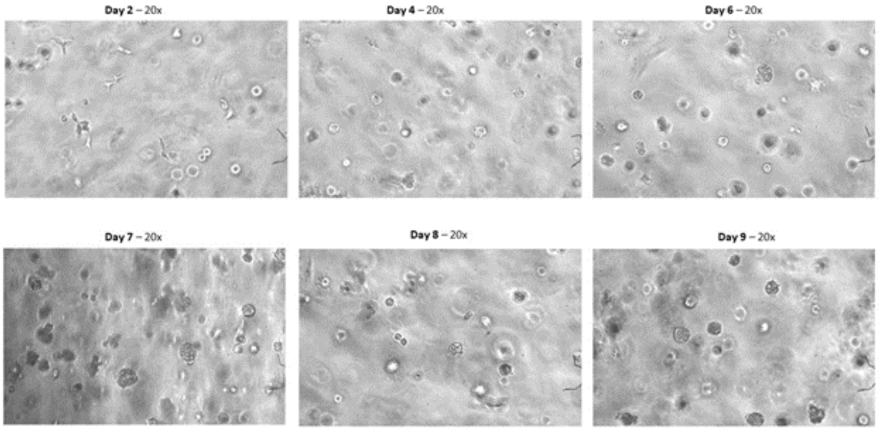

SKOV3 cells grown for 9 days in in GelTrex™. Spheroid formation by day 6 can be seen in 3D cultures.

Figure S2. Growth of SKOV3 cells from Day 2 to Day 9 showing clear spheroid-like structures.
